# Correlation Between Opioid Drug Prescription and Opioid-Related Mortality in Spain as a Surveillance Tool: Ecological Study

**DOI:** 10.2196/43776

**Published:** 2023-06-28

**Authors:** Alejandro Salazar, Soledad Moreno-Pulido, Pablo Prego-Meleiro, Jesús Henares-Montiel, José Pulido, Marta Donat, Gabriel Sotres-Fernandez, Luis Sordo

**Affiliations:** 1 Observatory of Pain University of Cádiz Cádiz Spain; 2 Biomedical Research and Innovation Institute of Cádiz (INiBICA), Research Unit Puerta del Mar University Hospital University of Cadiz Cádiz Spain; 3 Department of Statistics and Operational Research University of Cádiz Puerto Real Spain; 4 Department of Mathematics University of Cádiz Puerto Real Spain; 5 Department of Public Health and Maternal-Child Health Faculty of Medicine Complutense University of Madrid Madrid Spain; 6 Department of Analytical Chemistry, Physical Chemistry and Chemical Engineering Faculty of Pharmacy University of Alcalá Alcalá de Henares Spain; 7 Center for Biomedical Research in Epidemiology and Public Health (CIBER) Madrid Spain; 8 Andalusian School of Public Health Granada Spain; 9 National School of Health Salud Carlos III Health Institute Madrid Spain; 10 Oncology Department Hospital Quirón Salud Madrid Spain; 11 Department of Medicine Faculty of Biomedical and Health Sciences Universidad Europea de Madrid Madrid Spain

**Keywords:** opioid, overdose, drug overdose, opioid-related deaths, mortality, tramadol, fentanyl, substance use, substance misuse, substance abuse, ecological study, death

## Abstract

**Background:**

Opioid drug prescription (ODP) and opioid-related mortality (ORM) have increased in Spain. However, their relationship is complex, as ORM is registered without considering the type of opioid (legal or illegal).

**Objective:**

This ecological study aimed to examine the correlation between ODP and ORM in Spain and discuss their usefulness as a surveillance tool.

**Methods:**

This was an ecological descriptive study using retrospective annual data (2000-2019) from the Spanish general population. Data were collected from people of all ages. Information on ODP was obtained from the Spanish Medicines Agency in daily doses per 1000 inhabitants per day (DHD) for total ODP, total ODP excluding those with better safety protocols (codeine and tramadol), and each opioid drug separately. Rates of ORM (per 1,000,000 inhabitants) were calculated based on deaths registered (International Classification of Diseases, 10th Revision codes) as opioid poisoning by the National Statistics Institute, derived from the drug data recorded by medical examiners in death certificates. Opioid-related deaths were considered to be those that indicated opioid consumption (accidental, infringed, or self-inflicted) as the main cause of death: death due to accidental poisoning (X40-X44), intentional self-inflicted poisoning (X60-X64), drug-induced aggression (X85), and poisoning of undetermined intention (Y10-Y14). A descriptive analysis was carried out, and correlations between the annual rates of ORM and DHD of the prescribed opioid drugs globally, excluding medications of the least potential risk of overdose and lowest treatment tier, were analyzed using Pearson linear correlation coefficient. Their temporal evolution was analyzed using cross-correlations with 24 lags and the cross-correlation function. The analyses were carried out using Stata and StatGraphics Centurion 19.

**Results:**

The rate of ORM (2000-2019) ranged between 14 and 23 deaths per 1,000,000 inhabitants, with a minimum in 2006 and an increasing trend starting in 2010. The ODP ranged between 1.51 to 19.94 DHD. The rates of ORM were directly correlated with the DHD of total ODP (*r*=0.597; *P*=.006), total ODP without codeine and tramadol (*r*=0.934; *P*<.001), and every prescribed opioid except buprenorphine (*P*=.47). In the time analysis, correlations between DHD and ORM were observed in the same year, although not statistically significant (all *P*≥.05).

**Conclusions:**

There is a correlation between greater availability of prescribed opioid drugs and an increase in opioid-related deaths. The correlation between ODP and ORM may be a useful tool in monitoring legal opiates and possible disturbances in the illegal market. The role of tramadol (an easily prescribed opioid) is important in this correlation, as is that of fentanyl (the strongest opioid). Measures stronger than recommendations need to be taken to reduce off-label prescribing. This study shows that not only is opioid use directly related to the prescribing of opioid drugs above what is desirable but also an increase in deaths.

## Introduction

The prescription of opioid drugs has grown enormously in Spain [1,2], with an increase of over 300% since 2001 [3,4]. This is not a local phenomenon. The same is occurring in many countries in Europe and around the world, resulting in a serious public health problem [5-7].

The increase in the opioid drug prescription (ODP) in Spain has been highlighted and registered in pharmaceutical surveillance systems [8], in addition to occasional investigations. This has resulted in recommendations to increase the precision of both prescription indications and duration of treatment [9,10], aligned with what is occurring in the international context [11]. The use of these medications has helped many people to manage their illnesses [9,11], especially those with chronic conditions and cancer [12]. However, the use of these medications is related to the potential appearance of adverse effects: the 2 most relevant are the development of an addiction to these drugs and death due to overdose [1,13].

The opioid crisis in North America is related to the medical prescription of these drugs. Both the number of people addicted to medically prescribed opioids and those addicted to other illegal opioids have increased dramatically in the last decade. The consequences for health, the loss of life years, and the economic costs are enormous [14-16].

The current data on the situation in Spain do not show a situation similar to that of North America [1,17]. The increase in ODP has not yet been accompanied by the phenomenon seen in other countries. However, concerns about the situation are important, and surveillance has been heightened in new profiles of addicted users in treatment as well as in the possible indirect indicators of an increase in consumption. This consumption has increased in an unequal way, based on different active ingredients and indications for use. There is an especially relevant increase in extrahospital use of tramadol and interhospital use of fentanyl [2,8,18], and at the same time, non-oncological use accounts for nearly 90% in certain cases [19].

Together with the registry of ODP, one of the most valuable indicators of the growth and type of use is the registry of deaths due to opioid overdose, also referred to as opioid-related mortality (ORM) [13,20]. This indicator reflects the number of deaths whose principal cause is overdose of any opioid and excludes the deaths due to other causes (such as terminal cancers) in which opioid treatment could exist [1,15,21]. The relationship between ODP and ORM is complex, as in Spain, they are recorded without differentiating between the type of opioid (legal or illegal). Therefore, determining the relationship between ODP and ORM can provide key information both about deaths due to prescribed opioid drugs and, in an indirect way, deaths due to illegal opioid consumption. The objective of this study was to examine the correlation between ODP and ORM in Spain from 2000 to 2019 and discuss their usefulness as a surveillance tool.

## Methods

This was an ecological study using retrospective annual data (2000-2019) from the Spanish general population. Data were collected on people of all ages for ORM and ODP.

### Data Source

To determine ORM, population data were collected from 2000 to 2019 through the Spanish National Statistics Institute [22], which produces a report of “statistics of deaths according to the cause of death derived from the drug data recorded by medical examiners in their death certificates” [23], following the criteria established by the World Health Organization (WHO) in the International Classification of Diseases, 10th Revision (ICD-10) [24]. This statistic provides information on mortality according to the cause of death. This is similar to methodology used in previous studies [1]. From these data, and according to the ICD-10 codes [24], we retrieved information on ORM. Opioid-related deaths were considered to be those that indicated opioid consumption (accidental, infringed, or self-inflicted) as the main cause of death: death due to accidental poisoning (X40-X44), intentional self-inflicted poisoning (X60-X64), drug-induced aggression (X85), and poisoning of undetermined intention (Y10-Y14). These codes excluded deaths due to opioid overdose in the contexts of compassionate sedation and euthanasia in terminal cases, given that in these cases, the codes were included in the disease codes. The complete list of codes is shown in Multimedia Appendix 1. These codes were used in ways that are similar to what has been carried out in prior publications [21], which also coincides with the codes for death related to the use of opioids as used in later publications [16,25,26]. These causes do not differentiate by the type of opioid, whether in the Spanish or international context. Some of the cited codes (X40, X60, and X85) do not fully correspond to the operational definitions that exist in the Spanish context. For each year (from 2000 to 2019), we report data on the number of ORM and ORM rates per 1,000,000 inhabitants (quotient between the number of opioid-related deaths and the total population).

The data on prescribed opioid drugs were obtained via the Spanish Agency of Medicines and Health Products (*Agencia Española de Medicamentos y Productos Sanitarios* [AEMPS]). All of the drugs require a medical prescription or special permissions to be sold in pharmacies or dispensed by health centers. The AEMPS receives state-level information on all of the prescriptions written for all drugs that contain opioids and separates them by active ingredient. In 2019, the complete list was made up of morphine, hydromorphone, oxycodone, oxycodone and naloxone, pethidine, fentanyl, dextropropoxyphene, pentazocine, buprenorphine, codeine and paracetamol, codeine and acetyl salicylic acid, codeine and ibuprofen, tramadol and paracetamol, tramadol and dexketoprofen, and tramadol and tapentadol. Prescribed opioid drugs were studied both globally and individually, which is explained later in this article. The measurement unit used was dose per 1000 inhabitants per day (DHD).

### Statistical Analysis

Normal distribution was confirmed for the variables (ODP and ORM) using Shapiro-Wilk tests, and we observed that all of the variables followed a normal distribution (Multimedia Appendix 2). Subsequently, a description of the variables’ evolution over time was carried out. We carried out an analysis of the correlation between the annual rates of ORM and the DHD of the prescribed opioid drugs globally, excluding medications of the least potential risk of overdose and lowest treatment tier [27] (codeine and tramadol), and separately, based on the frequency of their use or their growth over recent years from the initial descriptive analysis. The relationship between ORM and ODP was analyzed as follows: (1) total ODP, (2) total ODP excluding codeine, (3) total ODP excluding codeine and tramadol, (4) tramadol, (5) fentanyl, (6) tapentadol, (7) oxycodone (unifying presentations with and without naloxone), and (8) morphine. All correlations were calculated using Pearson linear correlation coefficient. Statistically significant differences were observed when *P*<.05.

Finally, an analysis of the evolution of each variable was carried out across time, using time series. The relationship between the total evolution of the mortality rate and the DHD of the rest of the drugs was analyzed via cross-correlations with 24 lags and the cross-correlation function. Using this method provides knowledge of the correlations of mortality over time *t* (in a determined year) and the DHD of each opioid drug in time *t – k* (in annual spikes or intervals; in this case: 1 year prior, 2 years prior, etc). This could indicate a potential impact of the increase in a dose during year *t* on mortality in *t – k* years (1 year later, 2 years later, etc), without necessarily demonstrating a causal relationship.

Time evolution was studied during the whole period of the data, for the periods 2000-2011 and 2012-2019, and for the periods 2000-2015 and 2016-2019. The fragmentation of these periods corresponded to changes in the trend in deaths observed in 2010 and the alert made by health authorities in 2016 about the increase in the use of these medications as well as a call for their rational use [28], respectively.

The analyses were carried out using the Stata statistical package (version 16.1; StataCorp LLC) and StatGraphics Centurion 19 (StatGraphics Technologies, Inc).

### Ethics Approval

This study was a secondary analysis of data with no identifying information available to the researchers. It was approved by the institutional review board at the Hospital Clínico San Carlos (affiliated to Complutense University) on March 18, 2021 (approval 21/167-E).

## Results

**Figure 1:**
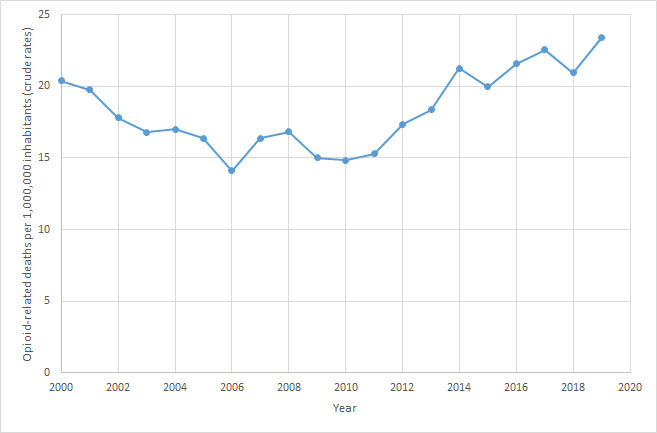
Evolution of opioid-related mortality per 1,000,000 inhabitants (2000-2019).

The temporal evolution of the rate of ORM from 2000 to 2019 ranged between 14 and 23 deaths per 1,000,000 inhabitants. Rates decreased progressively to a minimum in 2006, and after some fluctuations, there was a slight increase starting in 2010 that seemed to be stable over time (Figure 1).

**Figure 2:**
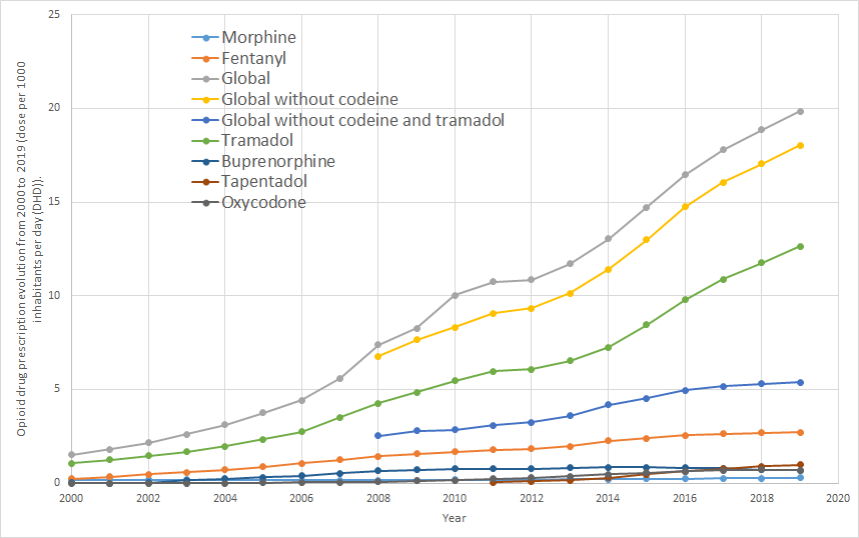
Opioid drug prescription evolution from 2000 to 2019 (unit: dose per 1000 inhabitants per day [DHD]).

In terms of the evolution of the DHD of the opioid drugs analyzed, we observed a continuous increase of more than 40% per year until 2006. Starting in 2006, this became more acute, especially in 2010 when the rate grew from 4.4 to 10 DHD, with a global increase from 2000 to 2019 of 1.51 to 19.83 DHD (12.6 times greater). The magnitude of tramadol stands out (prescribed alone or in conjunction with other nonopioid active ingredients); tramadol represents 63.7% of the total opioids prescribed, with an increase of 10% (of the total) since 2010. Codeine, however, declined from 17% of opioids in 2010 to 9% in 2019. Fentanyl accounted for 13% of prescribed opioids in 2019, followed by tapentadol (5%), buprenorphine (3.4%), and oxycodone (3%). Notable growth can be observed in all of these opioids. Fentanyl and tapentadol both grew by 1000% between 2000 and 2012, whereas oxycodone (first, without and, later, with naloxone) grew by 600% since 2011. The consumption of buprenorphine has been very stable over the past decade. Finally, morphine accounted for 1.3% of prescribed opioids, with an increase in consumption of 63% since 2010 (Figure 2).

**Figure 3:**
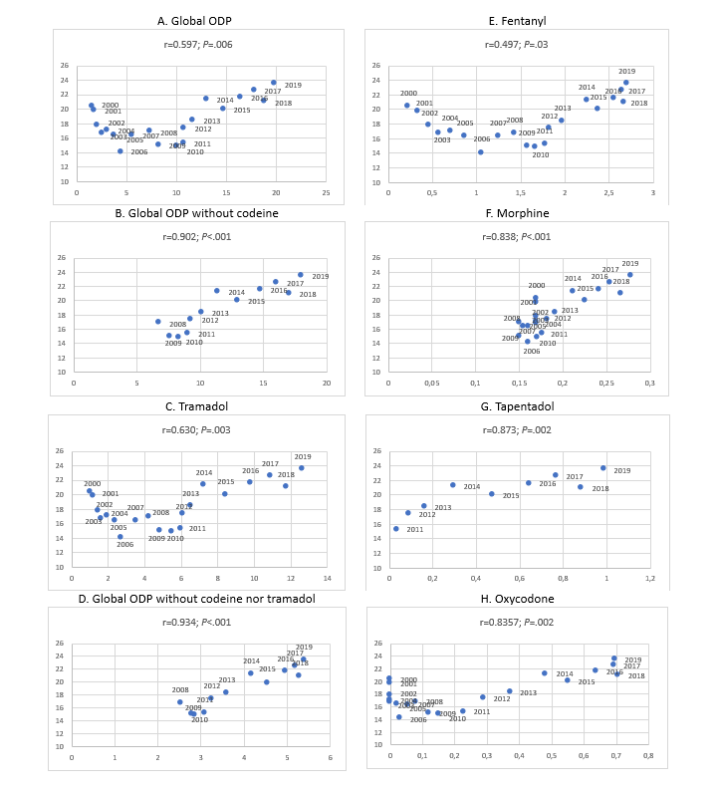
Correlation between opioid drug prescription (ODP; x-axis) and mortality rate per 1,000,000 inhabitants (y-axis).

Mortality rates for each year were directly correlated (the higher DHD of opioid drug, the higher the mortality rate) in all of the cases analyzed, except in the case of buprenorphine (*P*=.47). The strongest positive correlations were observed with global ODP without codeine (*r*=0.902; *P*<.001), global ODP (*r*=0.597; *P*=.006), global ODP without codeine or tramadol (*r*=0.934; *P*<.001), tapentadol (*r*=0.873; *P*=.003), and oxycodone (*r*=0.835; *P*=.002; Figure 3).

**Figure 4:**
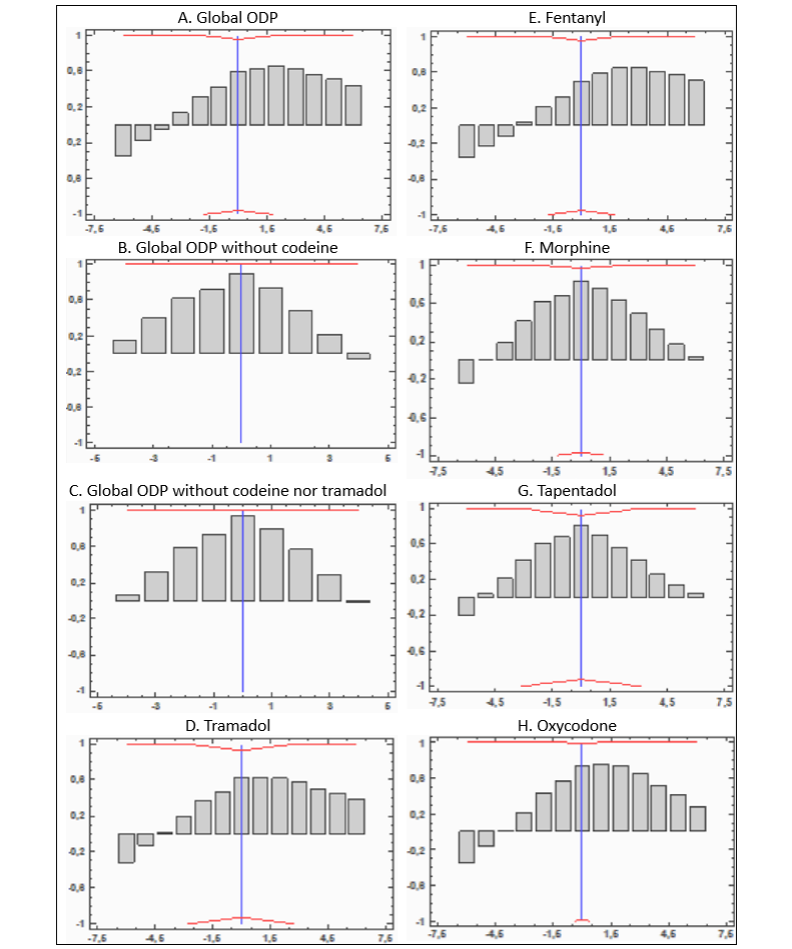
Cross-correlation between opioid-related mortality rates and opioid drug prescription (ODP). y-axis: correlation; x-axis: delay in years.

Figure 4 shows the cross-correlations between mortality rates and the DHD of the different drugs analyzed. In considering all of these drugs, the figure shows a greater correlation between prescribed DHD in a year and the mortality rate 3 years later, reaching a maximum in the second year. However, after eliminating prescribed drugs with codeine and those with codeine and tramadol from the totals, the correlations with the mortality rate can be observed for the same year and not so much for the following years. The same situation occurred when exclusively considering morphine and tapentadol, where the greatest correlations were observed in the same year and in the 2 following years, with a gradual dilution of the correlation in successive years. This same relationship can also be observed with tramadol alone or in conjunction with another medicine. Fentanyl seems to have an impact on mortality over the medium term, in the second or third years, whereas buprenorphine has a long-term impact of between 4 and 6 years. Despite this pattern, these differences were not significant (all *P*≥.05). The segmented analysis of the periods of 2000-2010 and 2011-2019 as well as 2000-2016 and 2017-2019 did not result in findings different from what is described here.

## Discussion

This study confirms the enormous increase in the prescription of opioid drugs in the last 2 decades in Spain. This increase was driven especially by tramadol, but fentanyl, due to its potency and hospital use, also plays a very relevant role. It also points out a correlation between the increases in ODP and ORM.

Considering the fact that illicit opioid use has decreased or remained the same in the period from 2001 to 2019 [17,20], this could suggest that deaths related to prescribed opioid drugs have increased.

The increase in ODP has been highlighted in the past by other studies carried out in Spain that use different data sources [2-4]. This study includes a larger geographic region, Spain’s national territory, in which medical indications are homogenous [28]. This suggests that the progression of ODP is evolving in a parallel way in different regions in Spain [4,17,18]. This increase corresponds with the situation in most other European countries [5].

Many of the causes of this enormous increase have been pointed out. The use of these drugs [11,12,28], as well as their target population, has increased (in large part due to the fact that the aging population experiences more disease and related pain [29]). However, both in Spain and in other countries where this phenomenon is taking place, prescriptions are increasing at a faster rate than these other factors [5,30,31]. More opioids are being prescribed and for longer terms than that recommended by treatment guidelines. This issue is beyond the scope of this study, but it is one in which the role of prescriptions, compared to other factors such as socioeconomic level or residing in rural versus urban areas [18], seems to be especially important given its potential as an area for improvement [32,33].

In addition to describing this general phenomenon, this study shows the correlation with ORM, which coincides with what has been shown to occur in other contexts [33]. In Spain, it appears that in the years included in this study, there has not been a proliferation in illegal consumption of prescribed opioid drugs or other opioids [17,20]. Therefore, it can be inferred that it is the prescribed opioid drugs and not illegal opioids that are provoking this increase in deaths. This suggestion should be taken with great prudence, but it corresponds with what has occurred in environments with greater problems related to illegal opioids [14]. With a lower proportion of illegal opioids, there is a greater correlation between prescriptions and opioid-related mortality. This study found the potential mortality from unstudied opioids to be minimal and the correlation between opioid medications and overdose deaths to be consistent with these occurrences. An absence of correlation might have indicated that illegal drugs were operating in the market. The results tell us that the increase in overdoses is related to an increase in opioid prescribing.

This fact reflects the situation up till 2019, but we should not forget that in the future, prescribed opioid drugs could spread to the illegal market, as has happened in other places [16,21,34]. Above all, precautions should be taken to detect such a situation if it were to occur [20]. The ORM-ODP correlation could be a useful indicator both separately and when harmonized with other indicators such as hospital admissions. In fact, from 2016 to 2019, hospital admissions related to poisoning, other consequences of external causes (S00-T88), and external causes of morbidity (V00-Y99) increased in Spain by 20% [35].

The existence of the ORM-ODP correlation may be as informative as its possible nonexistence in the future. The lack of a correlation could suggest that illegal opioids have increased [14] or that there has been an improvement in prescription practices. Furthermore, opioid-related deaths are an indicator of opioid consumption. It is the least desired adverse effect, but there is a proportionality between its frequency and consumption [20]. Therefore, the surveillance of ORM could provide information about possible variation in other parameters, such as addiction or other related comorbidities (associated infections, dual pathology, etc). Therefore, the surveillance of trends in ORM and ODP separately, as well as the correlations between them, could play an important role in the global monitoring of opioid consumption and its consequences.

In establishing preventive measures, it is useful to focus on certain active ingredients, in addition to prescribed opioid drugs in general, especially in the case of tramadol and fentanyl, in which we found an ORM-ODP correlation. These are 2 medications with different indications and safety profiles.

Tramadol accounts for not only the majority of prescriptions but its increase has also been constant and parallel to total ODP. It belongs to the group of second-tier analgesic opioids as defined by the WHO [27]. The indications of tramadol for non-oncological pain can possibly explain this increase, as suggested in other Spanish studies [18] and coinciding with the European and North American contexts [36]. It is unknown whether this increase corresponds to the increase in its target population. The indications for tramadol and the number of potential patients have increased [11,12]; however, studies of its misuse and misprescription are scarce and contradict each other [36-38]. This study did find a correlation between ORM and tramadol prescriptions; therefore, it is important to reinforce the correct use of tramadol among those who prescribe it [28], as well as to intensify surveillance. It is still the most prescribed opioid drug, where a simple prescription is all that is required [8]. It is much easier to prescribe than others.

In the third analgesic tier [27], fentanyl stands out due to its growth and the ORM-ODP correlation. It is the strongest opioid, specifically recommended for the treatment of irruptive oncological pain. It is also the opioid with the greatest potential number of overdoses, although this also depends on the posology [11,12,39]. This increase is in the context of the notable increase in the consumption of the strongest opioids [2-4,10]. This trend goes beyond the scope of this study. The number of prescriptions of opioids in the third tier increased by 9.6% between 2019 and 2020 [19]. What is surprising is that 89% is related to non-oncological indications [19]. Pain management is an essential component of medical care, but these data are worrisome [3]. Both the increase, as well as the possible adverse effects, have been highlighted in Spain by health authorities, who issued an alert in 2016 to restrain prescriptions to specific indications and the doses to the length of the disease [28]. This study did not find a change over time in the correlations prior to or after that date.

Of the opioid drugs studied, only buprenorphine was not correlated with ORM. It is possible that this could be due to the fact that this opioid is usually used as a substitute treatment for other opioids [40], with a stable number of users [20]. Data on the dispensation of buprenorphine did not differentiate between its use as an analgesic and as treatment for addiction, and as such, it is impossible to interpret these data.

In terms of the temporal patterns described, after eliminating the safest active ingredients from the totals (codeine and codeine with tramadol), the correlations with the mortality rate shifted and could be observed in the same year and less so in successive years. Individually, for morphine, tramadol, and tapentadol, the years with the greatest number of deaths coincided with the years with the greatest number of prescriptions of these drugs. Although these results are not statistically significant (probably because the time series analyzed is short and had few data points [19 data-year/opioid]), they could point to the fact that the ORM-ODP relationship is not mediated by all opioids. They should be studied individually, as they do not share the same indications, safety profiles, or prescription processes. Future studies should study in further depth the prescription of these drugs.

This study includes a number of limitations. Although the data collected correspond to precise definitions that use ICD-10 codes, the reliability of the determination and coding of the cause of death depends on each professional for each death and not on the data source itself. The codes selected are exclusively for deaths due to overdose. There are also specific codes for deaths provoked by opioids in euthanasia or compassionate sedation in the context of palliative care that we did not include. The deaths due to these other causes are not included in what we refer to as ORM. Even so, in some cases, it is possible to omit using the overdose code to avoid legal or administrative problems, especially in the case of accidents. Thus, the number of deaths could be understated. On the other hand, the data did not permit us to disaggregate by sex nor age, which could limit these conclusions. However, these data are acceptable in the international context, as the data source is used by the European Monitoring Centre for Drugs and Drug Addiction [41].

It is important to point out that the Spanish system exclusively includes the main cause of death, and not multiple causes. This differs from other information systems, such as the US system, in which several codes are collected. These codes can be cross-examined to refine the cases in which (1) opioid use has been registered and (2) a death associated with these substances has been recorded. This is the reason why we talk about opioid-related deaths rather than directly attributable causes of mortality in our paper.

This study suggests that at the population level, there is a correlation between greater availability of prescribed opioid drugs and an increase in opioid-related deaths. The correlation between ODP and ORM may be a useful tool in monitoring legal opiates but also possible disturbances in the illegal market. The role of tramadol (an easily prescribed opioid) is important in this correlation, as is that of fentanyl (the strongest opioid). Measures stronger than recommendations need to be taken to reduce off-label prescribing. This study shows that opioid use is not only directly related to the prescribing of opioid drugs above what is desirable but also an increase in deaths.

## Appendix

Multimedia Appendix 1 List of included codes.

Multimedia Appendix 2 Shapiro-Wilk normality test in prescribed opioid drugs.

## Abbreviations

AEMPS: Agencia Española de Medicamentos y Productos Sanitarios (Spanish Agency of Medicines and Health Products)
DHD: dose per 1000 inhabitants per day
ICD-10: International Classification of Diseases, 10th Revision
ODP: opioid drug prescription
ORM: opioid-related mortality
WHO: World Health Organization

## Data Availability

The Spanish population data [42] and the data on opioid-related mortality [43] are publicly available at the Spanish National Statistics Institute website.

The data prescribed opioid drugs were obtained via the Spanish Agency of Medicines and Health Products (*Agencia Española de Medicamentos y Productos Sanitarios* [AEMPS]) [44].
