# Supplementary material for: Correlation Between Opioid Drug Prescription and Opioid-Related Mortality in Spain as a Surveillance Tool: Ecological Study
Source: JMIR Public Health Surveill. 2023 Jun 28;9:e43776. doi: 10.2196/43776 (PMC10365608; doi:10.2196/43776)
Supplement: Multimedia Appendix 1 [file publichealth_v9i1e43776_app1.docx]

**ANNEX 1: List of included codes:**

X40 Accidental poisoning by, and exposure to, non-narcotic analgesics, antipyretics and antirheumatic drugs.

X41 Accidental poisoning by, and exposure to, anti-epileptic, sedative, hypnotic, antiparkinsonian and psychotropic drugs, not elsewhere classified.

X42 Accidental poisoning by, and exposure to, narcotics and psychodysleptics [hallucinogens], not elsewhere classified.

X43 Accidental poisoning by, and exposure to, other drugs acting on the autonomic nervous system.

X44 Accidental poisoning by, and exposure to, other drugs, medicaments and biological substances, and those not specified.

X60 Intentionally self-inflicted poisoning by, and exposure to, non-narcotic analgesics, antipyretics and antirheumatic drugs.

X61 Intentionally self-inflicted poisoning by, and exposure to, anti-epileptic, sedative, hypnotic, antiparkinsonian and psychotropic drugs, not elsewhere classified.

X62 Intentionally self-inflicted poisoning by, and exposure to, narcotics and psychodysleptics [hallucinogens], not elsewhere classified.

X63 Intentionally self-inflicted poisoning by, and exposure to, other drugs acting on the autonomic nervous system.

X64 Intentionally self-inflicted poisoning by, and exposure to, other drugs, medicaments and biological substances, and those not otherwise specified.

X85 Assault with drugs, medicaments and biological substances.

Y10 Poisoning by, and exposure to, non-narcotic analgesics, antipyretics and antirheumatic drugs, of undetermined intention.

Y11 Poisoning by, and exposure to antiepileptic, sedative, hypnotic, antiparkinsonian and psychotropic drugs, not elsewhere classified, of undetermined intention.

Y12 Poisoning by, and exposure to, narcotics and psychodysleptics [hallucinogens], not elsewhere classified, of undetermined intent.

Y13 Poisoning by, and exposure to, other drugs acting on the autonomic nervous system, of undetermined intent.

Y14 Poisoning by, and exposure to, other drugs, medicines and biological substances, and those not elsewhere specified, of undetermined intent.
