# Supplementary material for: Correlation Between Opioid Drug Prescription and Opioid-Related Mortality in Spain as a Surveillance Tool: Ecological Study
Source: JMIR Public Health Surveill. 2023 Jun 28;9:e43776. doi: 10.2196/43776 (PMC10365608; doi:10.2196/43776)
Supplement: Multimedia Appendix 2 [file publichealth_v9i1e43776_app2.docx]

ANNEX 2. Shapiro-Wilk normality test in prescribed opioid drugs. Ecological study in Spain.

| Variable | W | p-value | Normality |
| --- | --- | --- | --- |
| Global opioid drug prescription (ODP) | 0.915 | *.35* | Yes |
| **Global ODP w/o codeine** | 0.921 | *.40* | Yes |
| Global ODP w/o codeine nor tramadol | 0.897 | *.23* | Yes |
| Morphine | 0.935 | *.53* | Yes |
| Fentanyl | 0.879 | *.15* | Yes |
| Buprenorphine | 0.961 | *.81* | Yes |
| Morphine and tramadol | 0.912 | *.33* | Yes |
| Tramadol | 0.923 | *.12* | Yes |
| Tapentadol | 0.929 | *.47* | Yes |
| Oxycodone | 0.879 | *.046* | No |
